# Brief oral health promotion intervention among parents of young children to reduce early childhood dental decay

**DOI:** 10.1186/1471-2458-13-245

**Published:** 2013-03-20

**Authors:** Peter Arrow, Joseph Raheb, Margaret Miller

**Affiliations:** 1 WA Dental Health Services, 43 Mt Henry Road Como, 6152 Western Australia, and the Australian Research Centre for Population Oral Health, University of Adelaide, 122 Frome Street, Adelaide, 5000, Australia; 2 Department of Dental Hygiene and Therapy, Curtin University, C/ Oral Health Centre of Western Australia, 17 Monash Avenue, Nedlands, WA, 6009, Australia; 3 Child Health Promotion Research Centre, Edith Cowan University, 2 Bradford Street, Mt Lawley, Western Australia , 6050, Australia

**Keywords:** Motivational interviewing, Anticipatory guidance, Early childhood dental decay, Oral health promotion

## Abstract

**Background:**

Severe untreated dental decay affects a child’s growth, body weight, quality of life as well as cognitive development, and the effects extend beyond the child to the family, the community and the health care system. Early health behavioural factors, including dietary practices and eating patterns, can play a major role in the initiation and development of oral diseases, particularly dental caries. The parent/caregiver, usually the mother, has a critical role in the adoption of protective health care behaviours and parental feeding practices strongly influence children’s eating behaviours. This study will test if an early oral health promotion intervention through the use of brief motivational interviewing (MI) and anticipatory guidance (AG) approaches can reduce the incidence of early childhood dental decay and obesity.

**Methods:**

The study will be a randomised controlled study with parents and their new-born child/ren who are seen at 6–12 weeks of age by a child/community health nurse. Consenting parents will complete a questionnaire on oral health knowledge, behaviours, self-efficacy, oral health fatalism, parenting stress, prenatal and peri-natal health and socio-demographic factors at study commencement and at 12 and 36 months. Each child–parent pair will be allocated to an intervention or a standard care group, using a computer-generated random blocks. The standard group will be managed through the standard early oral health screening program; “lift the lip”. The intervention group will be provided with tailored oral health counselling by oral health consultants trained in MI and AG.

Participating children will be examined at 24, and 36 months for the occurrence of dental decay and have their height and weight recorded. Dietary information obtained from a food frequency chart will be used to determine food and dietary patterns. Data analysis will use intention to treat and per protocol analysis and will use tests of independent proportions and means. Multivariate statistical tests will also be used to take account of socio-economic and demographic factors in addition to parental knowledge, behaviour, self-efficacy, and parent/child stress.

**Discussion:**

The study will test the effects of an oral health promotion intervention to affect oral health and general health and have the potential to demonstrate the “common risk factor” approach to health promotion.

**Trial registration:**

Australian New Zealand Clinical Trials Registry: http://ACTRN12611000997954

## Background

There is little information available on the prevalence of early childhood dental decay in Australia. The most recent report on Australian children’s dental health indicated that, among 4-year-olds in 2005, approximately 37 percent of children had at least one decayed baby tooth. On average, there were 1.84 decayed, missing or filled baby teeth (dmft), of which 84 percent comprised of untreated dental decay [1]. In another study, parental report of dental decay experience (cavities, fillings or extractions) among preschool children (aged 2–3 years) was approximately 3% nationally with substantial variation between the States and Territories [2].

Information available from the Western Australian Dental Health Service (DHS) suggests that the majority of children that register with the Service at age five had not attended any dental care facility and were unlikely to have received any specific oral health care information. The mean decay experience (dmft) of WA 5-year-olds in 2009 was 1.26 of which 1.03 (82%) was the “d” component (untreated dental decay) (personal communication: DHS, 2009). One in three 5-year-old children examined in WA was affected by dental decay and one in ten had more than five teeth affected with dental decay, the majority of which was untreated. Severe untreated decay affects a child’s growth, body weight, quality of life as well as cognitive development, and the effects extend beyond the child, to the family, the community and the health care system [3-5].

Data from elsewhere suggest that the decay experience among children is increasing in Australia. A report from South Australia (SA) showed that, for children attending the School Dental Service in SA, the decay experience among 6–7-year-olds in 1998 was 1.16 and nearly doubling to 2.29 in 2008; and among 5-year-olds the decay experience in 2008 was 1.89 of which 1.2 was untreated decay, and 44% of the children were affected with decay [6].

The management of dental disease for this younger age group is demanding and the severity of the condition often necessitates comprehensive care under general anaesthesia, which is costly and is not without risk [7,8]. The cost of hospital admissions for dental conditions among children in WA was approximately $10 million per annum over the four-year period of 1999–2003 [9]. The 1–4 year-old age group comprised 25% of the admissions and had the highest rate for dental admissions related to dental decay and associated pathology due to dental decay (pulp and peri-apical tissue conditions). In a study using linked data from various agencies in WA, 76% of children aged less than 5 years who were admitted to a hospital for dental conditions were admitted for diseases of the hard tissues of teeth, and the proportion of children admitted for dental conditions over the period studied (birth years 1980–1995) doubled [10]. Dental disease causes a lot of pain and suffering and its treatment is expensive, consuming between 4%–11% of the health budgets of developed nations and costs more than the treatment of cardiovascular disease, cancer and osteoporosis [11]. In Australia, there is a trend of increasing admissions for dental treatment among children [12].

Current understanding of dental decay is that it is largely preventable and personal behaviours, including eating patterns and dietary practices, can have significant effects on oral health outcomes. The conceptual model developed by Fisher-Owens et al. provides a framework for guiding an understanding of the wider influences on child oral health and the factors contributing to early childhood decay [13]. For the young child, the wider influences on child oral health, the family, and in particular the mother, have a major influence on food preferences and dietary practices. Furthermore, there is a recognition of the importance of early interventions to affect appropriate dietary and nutritional practices [14,15].

There is also a further recognition that oral diseases share risk factors with other chronic diseases and conditions, such as heart disease, cancer, strokes, diabetes and obesity [16]. World-wide and in Australia, a diet-related health concern among children is that of childhood obesity, and in Australia 23% of children aged between 2 and 16 years are overweight or obese [17,18]. Childhood overweight and obesity has a strong association with premature mortality and cardiometabolic morbidity in adulthood [19,20]. A life-course approach to chronic disease development posits the importance of early childhood factors in the development of chronic ill-health, including oral diseases [21].

There is a strong association between dietary factors and oral disease, in particular with dental decay [22], and there are associations between early childhood dietary factors and childhood obesity [23]. Furthermore, an association has been shown between early childhood dietary patterns and dental decay in adolescence and an association between adiposity at adolescence with dental decay [24]. The sharing of risk factors between oral and general health, and the effects of early childhood factors on the later development of diseases points to practical and economic reasons for integrating oral health promotion efforts that can achieve multiple aims during early childhood. For example, the role that periodontal treatment has in glycaemic control among diabetic patients has been highlighted [25] and, general health promotion for positive early childhood nutrition has shown benefits for oral health [26]. Interventions in early childhood are being tested to reduce the prevalence of early childhood obesity [15,27], although effects on oral health have not been reported.

The reported findings in this brief review suggest that the problem of early childhood dental disease requires substantial resources and interventions at a number of levels to manage, and the problem appears to be increasing. The intertwining of risk factors for dental decay and other chronic conditions suggests that health promotion interventions for dental decay may also have effects on other chronic health conditions.

Traditional dental health education approaches have had limited success in preventing dental decay [28]. Anticipatory guidance (AG) is a model of health promotion where practical information appropriate for the developmental stage of the child is provided to parents in anticipation of significant milestones for the child. The information acts as a sign-post for parents to anticipate the forthcoming changes and facilitate action to maximise the child’s potential and meet its needs [29]. The use of AG in oral health promotion, where information on oral health care and nutrition was provided to expectant mothers, and again when the child was 6 and 12 months of age, has shown positive results in the prevention of early childhood decay in an Australian setting [30].

The motivational interviewing (MI) approach, which has been used in many different settings, relies on a brief empathic counselling session where the client is helped to explore and verbalise the reasons for changing the health behaviour and to find the reasons for the change themselves [31]. The impetus for change stems from the person and is not imposed by the counsellor; the client is the initiator and an active participant in the change process and not merely a recipient of information being delivered by an expert [32]. The use of the MI approach, underpinned by stages of change theory has shown promise in reducing early childhood decay [33-35]. A recent systematic review identified MI as being the most effective approach for altering health behaviour in an individual oral health promotion setting [36]. The effectiveness of MI may be enhanced when it’s combined with other interventions and a combined AG and MI approach may have additive effects [31].

It is hypothesised that the effects on a child’s oral health will be mediated through the primary caregiver. Hence, we will also collect information to test the potential mediators of early childhood caries [37], in particular, psychosocial measures comprising oral health-related self-efficacy, knowledge about appropriate baby bottle use and children’s oral hygiene and belief in oral fatalism based on that operationalised by Finlayson et al. [38,39]; parental stress [40] and social support [41] of the parent/caregiver.

The proposed study will use a combined brief MI/AG approach with the parents of young children aimed at reducing early childhood decay and obesity among children. The aims of the study are to:

1. *Measure the baseline oral health knowledge, behaviour, attitudes, self-efficacy of parents/caregivers and parental stress levels of new-born children and then to measure and compare the change in these factors among the intervention and control groups at 12 and 36 months.* These factors are expected to be influential in affecting the oral health of children. The hypothesis tested is that early oral health promotion intervention will change the oral health knowledge, behaviour, attitudes, self-efficacy and parental stress levels to achieve improved oral health*.*

2. *Measure and compare the occurrence of early childhood dental decay among the intervention and control groups.* The hypothesis tested is that early health promotion will reduce the incidence of dental decay.

3. *Measure and compare the consumption of cariogenic/obesogenic foods and drinks and the growth and development of the child study participants.* The hypothesis tested is that early childhood oral health promotion will reduce consumption of cariogenic/obesogenic foods and the prevalence of early childhood adiposity.

4. *Compare the decay experience and increment and childhood adiposity among children in the intervention and control groups when the children are older, at pre-primary school age (2 years post active intervention, approximately 5 years of age).* The hypothesis being tested is that early intervention with AG and MI will have longer lasting effects beyond the immediate intervention in early childhood and the benefits are retained into the period when the permanent teeth are becoming established.

## Methods

The study is a randomized controlled trial to test an oral health promotion intervention aimed at new-born children and their parents/carers in metropolitan Perth (population 1.7 million) and the regional cities of Bunbury (population 32,000) and Busselton (population 31,000) in Western Australia. It is a collaboration between Child and Adolescent Community Health and Dental Health Services (DHS), both Department of Health state-wide agencies delivering services for children.

Study participants will be recruited from all child/community health clinics in metropolitan Perth (fluoridated; 0.8 mg/L fluoride) and Bunbury/Busselton (non-fluoridated; < 0.2 mg/L fluoride). Children/parent dyads attending the child health clinics from these locations will be invited to participate, and the randomisation procedure will be based on individual randomisation stratified on the basis of residential location of Perth or Bunbury/Busselton. All new-born WA children are referred to their local child health clinic and receive a home visit from the child health nurse within 6 weeks of birth and are seen at the local child health clinics at regular intervals for health assessments throughout the early years. Parents of a new-born child presenting to selected child health clinics will be invited to participate and provided with information about the study including an oral health promotional package, a consent form and a self-complete questionnaire by the child health nurse. Upon receipt of consent to participate, consenting parent/caregiver and child dyads will be randomly allocated to either an intervention or control group by a central study coordinator using a computer generated random permuted blocks (block sizes; 4, 6, 8, 10, 12). The blocks will ensure that at most, an imbalance between test and control groups will be 6 participants at the conclusion of participant recruitment. Blinding of parent participants and counsellors will not be possible.

The self-completed questionnaire will collect socio-demographic (age, sex, education level, family income, Aboriginality) and psychosocial information (oral health-related self-efficacy, knowledge about appropriate baby bottle use and children’s oral hygiene and belief in oral fatalism, instrumental social support and parental stress) and parental perceptions of treatment need and oral health care behaviours. The psychosocial measures have been used in other studies and have had their psychometric properties validated [38,39].

The psychosocial measures are shown in List of Psychocosial Measures. Oral health self-efficacy comprised nine items scored on a Likert scale from 1 (not at all confident) to 4 (very confident); higher scores indicate greater self-efficacy. Oral health knowledge on appropriate use of baby bottle and children’s oral hygiene comprise 10 items scored on a Likert scale from 1 (strongly agree) to 5 (strongly disagree); higher scores indicate better knowledge. Oral health fatalism comprised one item, “Most children eventually develop dental cavities”, scored 1 (strongly agree) to 5 (strongly disagree). The parenting stress instrument comprised six items scored on a Likert scale, 1 (never) to 5 (almost always); higher scores indicate higher levels of stress. The social support instrument comprised four items scored “yes” or “no”. Mean scores from sum of the item scores will be used for the oral health self-efficacy, oral health knowledge and parenting stress scales while the oral health fatalism score will be converted to a binary variable reflecting parent/caregiver agreement with the statement and each item of the instrumental social support instrument will be  presented as frequency scores and used as a binary variable.List of Psychological measures 

Oral Health Self-efficacy; scale, 1 = not at all confident, 4 = very confident

How confident do you feel that you are able to make sure that your child’s teeth are brushed before bedtime when you are:

1. Under a lot of stress?

2. Depressed?

3. Anxious?

4. Feeling like you do not have time?

5. Tired?

6. Worrying about other things in your life?

7. Bothered by your child crying?

8. Bothered because your child doesn’t stay still when you want him or her to brush?

9. Told by your child that he/she does not feel like brushing right now?

Knowledge of bottle use; scale, 1 = strongly agree, 5 = strongly disagree

1. Putting a baby to bed with a bottle helps the child to be better fed.

2. Putting a baby to bed with a bottle helps the child sleep better.

3. Putting a baby to bed with a bottle helps the child to gain weight and grow.

4. There is nothing wrong with putting the baby to bed with a bottle.

Knowledge of children’s oral hygiene; scale, 1 = strongly agree, 5 = strongly disagree

1. Cavities in baby teeth don’t matter since they fall out anyway.

2. Keeping baby teeth clean is not very important; after all, they fall out.

3. There is not much I can do to stop my child from developing dental cavities.

4. There is not much I can do to help my child have healthy teeth.

5. Children don’t need to brush everyday until they get their permanent teeth.

6. Children don’t really need their own toothbrush until all their teeth come in.

Oral health fatalism; scale 1 = strongly disagree, 5 = strongly agree

1. Most children eventually develop dental cavities.

Parental stress; scale 1 = never, 5 = almost always

1. How often do you feel that you have too little time to spend by yourself?

2. How often do you wish you did not have so many responsibilities?

3. How often would you say your child (or children) gets on your nerves?

4. How often do you feel that your child/ren is/are making too many demands on you?

5. How often do you feel that being a parent is much more work than pleasure?

6. How often do you feel tired, worn out, or exhausted from raising a family?

Instrumental support; scale “yes”, “no”

If you need to, is there someone you can count on to……

1. ……Run errands for you?

2. ……Lend you money?

3. ……Watch over your child/ren?

4. ……Lend you their car or give you a lift?

We will also seek information relating to the mother’s health during the last trimester of pregnancy and the child’s health in the peri-natal period at base-line. The weight and height of each child and primary caregiver will be measured at 24- and 36- month follow-up visits. Parents will recall 24-hour and usual dietary information for each child using a self-administered short food and beverage frequency questionnaire at the 12- and 36-month follow-ups. The 12- and 36-month questionnaire will also collect information relating to oral health care behaviours for the child including toothbrushing and use of fluoride toothpastes, fluid consumption, and use of professional oral health care services.

Dietary related information collected via a 24 hour recall questionnaire will be coded for cariogenic potential of the foods and drinks using the approach outlined by Lee and Messer (2010) [42] and the approach outlined by Bennet et al. (2009) for obesogenic potential of the foods and drinks [43].

The intervention group will be provided with oral health promotion delivered by a trained dental assistant (oral health counsellor, OHC) using the MI/AG protocol. The training of the counsellors involved a workshop comprising two days of didactic presentations, role-playing exercises and a practice counselling session with a parent–child dyad at the DHS’ School Dental Therapy training facility. An evaluation of the training program was undertaken and consisted of a MI pre- and post-test knowledge–base questionnaire, a Helpful Response Questionnaire and a trainer evaluation questionnaire. The knowledge and trainer evaluation questionnaires were based on those developed by Yale University School of Medicine [44]. The Helpful Response Questionnaire utilised was developed by Miller et al. [45].

At this stage, preliminary analyses have been undertaken of the OHCs’ knowledge of MI and trainer evaluation. The questionnaire to assess the OHCs' knowledge of the principles and practices of MI was administered as a pre–test (immediately before training) and post–test (immediately after training). It consisted of ten multiple-choice questions. OHCs scored one point for each correct answer.

From a maximum possible score of 10, the OHCs’ knowledge pre–test mean score was 4.3 (SD = 1.49) and post–test score was 6.4 (SD = 1.43). Comparison of mean scores using a paired two–sample t–test revealed a statistically significant difference *(p = 0.01).* It was concluded that the two-day training workshop increased OHCs’ knowledge of MI principles and practices. Full details of the evaluation of the training workshop for OHCs will be presented elsewhere (Manuscript in preparation).

Monitoring, feedback and improvement of the OHCs’ competence in the use of MI is on-going through the analysis of audio recordings.

Counsellors will be further assisted and supported by the researchers listening to selected interview sessions to monitor the fidelity of the MI/AG approach. Three, one-day follow-up meetings with the counsellors was also undertaken to further develop and refine the MI/AG counselling approach. Throughout the research period the fidelity of the MI intervention will be monitored with audio recordings, which will later be analysed using the Motivational Interviewing Treatment Integrity (MITI) instrument developed by Moyers et al. (2005) [46]. The MITI is a behavioural coding system to measure competence in the use of MI and enable the provision of feedback about MI practice. The MITI consists of two components: the global scores and the behaviour counts. Using the global score system, the coder assigns a number along a seven-point Likert scale to rate the dimensions of interviewer empathy and MI spirit. The behaviour count requires the coder to tally instances of interviewer behaviours relating to giving information, questioning, reflection, MI adherent and non-adherent skills. Global scores and behaviour counts are then compared with recommended thresholds in the MITI coding manual (Moyers et al. 2003) [47]. This approach enables OHCs to be provided with structured, formal feedback about ways to improve MI practice.

The OHCs employed in this intervention are fully qualified and experienced Dental Therapists and Dental Clinic Assistants. They receive education in basic oral health messages as part of their studies and through regular in-service training that their employer DHS conducts. In addition, comprehensive background notes on early childhood oral health promotion and AG were provided to the OHCs.

The control group will receive the standard care delivered through the universal “lift the lip” program (available throughout WA since 2011). The “lift the lip” protocol requires a child who presents to a child/community health clinic for a review at 8, 18 and 36 months of age to be screened for signs of dental decay by a child/community health nurse. An inspection of the oral cavity is undertaken, usually by lifting the upper lip and inspecting the upper anterior teeth for signs of decay. If signs of decay are detected, the parent is offered a referral to a dental practitioner, either to a private practitioner, or to a government general dental clinic if eligible for government supported dental care. All children, test and control, will continue to be provided with the standard lift the lip screening throughout the study period.

### Procedures

The OHC will undertake the first counselling session (home visit, expected to last up to 30 minutes) within 4 weeks after consent with two additional counselling sessions, negotiated between the OHC and the study participant as to face-to-face or phone contact, in the first 6 months (expected to last 15–30 minutes). The protocol for MI/AG will be adapted from Weinstein et al. and Plutzer & Spencer [30,34].

The MI/AG approach will follow a structured approach of rapport establishment and identification of oral health and nutritional needs using empathic reflective listening; presentation of menu of options and information with permission; discussion of options and elicitation of “change talk”; elicitation of importance and confidence in behavioural change; the development of behaviour change plan; and a schedule of follow-up. All discussions will be undertaken in a collaborative, person centred approach central to the MI “spirit”. Whilst the menu will present a range of options with potential to affect early childhood dental decay, the discussion will be limited to one or two topics of primary concern to the study participant, in keeping with the MI approach. Negative responses during the counselling session will be accepted without rebuttal and issues left for exploration at a subsequent session. AG will follow the anticipated milestones of teething and transition from wholly breast- or formula-fed to solid foods. Again, topics for discussion will arise in conversation with the study participant with guidance from the counsellor and the counsellor will provide information only when requested to do so. The information will incorporate appropriate dietary and nutritional guidance, following the national guidelines, to assist in the development of healthful behaviours, with the counsellors linking oral and general health impacts of these behaviours [48]. Review of audio recordings of counselling sessions will be undertaken to monitor protocol compliance.

### Sample size and power

Sample size of approximately 514 children in each arm of the trial is required to detect a 35% reduction in dental decay experience [based on decay experience of children within DHS; dmft 1.26 (SD 2.63), test dmft 0.8] with a probability of 0.8 (power 80%) and rejecting the likelihood of no difference at probability 0.05 (α = 0.05) [49]. This is a conservative sample allowance to detect a difference, since an Australian intervention study on early childhood decay found 79% reduction in decay incidence among 20 month-old children [23]. Allowing for drop-off and loss to follow-up over the following 3 years, an initial sample of approximately 750 in each group of the trial would be required (an earlier study of caries prevention within SDS found attrition rate of 9% per annum over two years [50]). The estimated sample size will have a power of 94% to detect a 35% reduction in the prevalence of early childhood decay (dmft > 0; from 0.25 to 0.16) between the groups (α = 0.05). The estimated sample size will also permit detecting a 33% difference in prevalence of overweight or obese children between the two groups (from 20% to 14%) with 80% power and α = 0.05.

**Figure 1:**
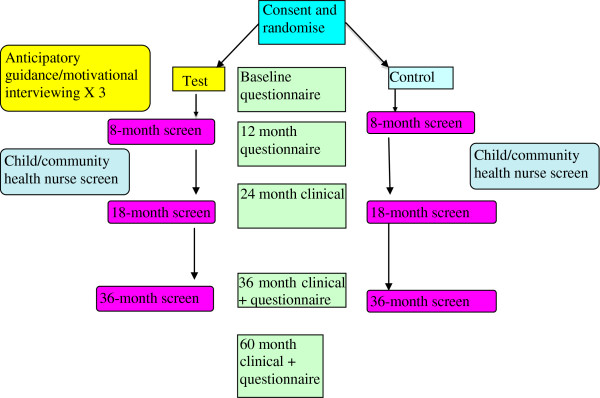
Study participant’s flow chart.

Ethical approval for the study has been provided by the University of Western Australia Human Research Ethics Committee (RA/4/1/4469), the Princess Margaret Hospital for Children Ethics Committee (Reg. No. 1900/EP), and the WA Country Health Services Ethics Committee (ref 2011:05). Figure 1 shows the study sample and pathway for the study participants in the test and control arms. All child participants will have the same clinical and other information collected again at approximately 5 years of age. Sample maintenance strategies, including card mail-outs on the child’s birthday, change of address notification and regular updating of the study will be undertaken.

### Measures

The primary outcome measures are the incidence of dental decay in primary teeth and the prevalence of obesity. Children will be examined at the dental therapy clinics of the DHS of Western Australia for the incidence of dental decay by two blinded, calibrated examiners at 24 and 36 months of age, using the criteria specified by Workshop on Reporting Early Childhood Caries [51] and at 60 months using the criteria of the WHO [52].

Examiner training will involve examination of early childhood decay under the guidance of a specialist paediatric dentist. Further examiner calibration will be undertaken by examination of 10 children consenting to participate in an oral screening program of DHS. During field examinations, approximately 5% of study participants will be re-examined to test for intra- and inter-examiner reliability. Child height/length and weight will be measured and Body Mass Index calculated (BMI; weight/height^2^) and BMI z score determined at 24 and 36 months, using the age-specific WHO growth standards [53]. Number needed to treat as an effect measure of the program will be estimated [54]. Examiner training will be repeated for each cycle of the clinical examinations to maintain reliability over the course of the study.

Secondary outcome measures will be changes in knowledge, attitude, behaviour and self-efficacy of parents towards the oral health of their child and differences in nutritional and dietary patterns between groups. Long-term outcomes will be the incidence of dental decay and referral for care under general anaesthesia among the groups when the child is 5 years of age.

### Data protection

The research will adhere to guidelines within the Australian Code for the Responsible Conduct of Research [55]. All collected data will be transcribed into electronic format and stored on a password-protected computer accessible only by the researchers. A separate electronic file will be created, excluding identification data for data analysis.

### Data analysis

Oral health outcomes will be defined by counts of decayed, missing and filled teeth/surfaces and include non-cavitated lesions. Predictor variables will be on the basis of group allocation to intervention or control group and the posited psychosocial mediating variables will be defined using the approach adopted by Finlayson et al. [39].

Appropriate parametric and non-parametric statistics will be used to test the aims of the study. Differences in outcomes between the groups will be tested on an intention to treat and per protocol basis. Univariate analyses using t-tests for continuous variables (*oral health knowledge, behaviour, attitudes, self-efficacy and parental stress of parents/caregivers*), and Chi square analyses for categorical variables will be undertaken to examine the baseline factors and their association with group assignment. Univariate analyses will also be used to examine the association between the primary outcomes of dental decay incidence and prevalence, and early childhood obesity prevalence, with group allocation.

Multivariate analysis will be used to control for potential confounding factors to examine the effects of treatment strategies, and will use linear regression for continuous outcome variables and test for mediator effects using the approach outlined by MacKinnon and Luecken (2011) [56], logistic regression for binary outcomes (dental decay prevalence, obesity prevalence) and Poisson regression to estimate rates of disease occurrence. Site-specific measures of caries incidence within an individual will use GEE analysis to control for correlation within an individual. Effect size will be estimated through calculation of incidence density ratios for dental decay and prevalence ratios for obesity. Effect measures will also be presented through calculation of number needed to treat and its associated 95% confidence interval. The statistically significant level will be at α = 0.05.

## Discussion

The expected benefits from the interventions are improved oral health knowledge, behaviours, and self-efficacy of parents/caregivers in the short term. Long-term benefits are improved oral health status of children with reduction in dental disease experience and where treatment is required it will be minimal and reduce the need for management under general anaesthesia and at a lower cost for care. There will also be positive outcomes in preventing early childhood obesity. Research findings will be translated into policy and practice and will build on existing cross-sector links between Child and Adolescent Community Health and DHS. The study will also develop teaching/training modules for primary health care personnel and undergraduate teaching. In addition to evaluating the workshop undertaken by the OHCs and on-going monitoring, we will administer the post-test to the counsellors at the end of the intervention period to enable an assessment of the sustained effectiveness of such a training program.

This study will provide evidence for the capacity to provide training to non-clinical oral health personnel to undertake primary oral health promotion within a community. It will also test the efficacy of using non-clinical oral health personnel provided with training to provide oral health counselling using a community-based approach to reduce the incidence of early childhood dental decay. The approach has the potential to improve oral health outcomes for young children utilising an established service delivery procedure (home visits by child/community health nurses to support parents with a new-born child) with significant potential for improved cost-effectiveness in the delivery of oral health promotion within a community. The study will also support the recognition of the impact of oral health conditions among the pre-school child beyond the child, and for the development of oral health policy for a relatively neglected group within the community in Australia.

The study will also test the common risk factor approach to chronic disease prevention, and the complementary role that oral health promotion can play in promoting general health, in particular reducing the growing prevalence of childhood obesity.

## Abbreviations

BMI: Body Mass Index; DHS: Western Australian Dental Health Services; dmft: Count of decayed missing, and filled deciduous teeth; GEE: Generalized Estimating Equation; MI/AG: Motivational interviewing/anticipatory guidance; SA: South Australia; WA: Western Australia; WHO: World Health Organization

## Competing interests

The authors declare that they have no competing interests associated with this research.

## Authors’ contributions

PA conceived and designed the study, undertook ethics applications, participated in training the field staff, drafted the manuscript, and coordinated the data collection and data management. JR provided intellectual input into the study design, undertook field staff training, participated in data collection and in manuscript preparation. MM provided intellectual input into the study design and assisted in the development of the dietary questionnaire and in manuscript preparation. All the authors were involved in revising the manuscript and provided intellectual input and read and approved the final manuscript.

## Pre-publication history

The pre-publication history for this paper can be accessed here:

http://www.biomedcentral.com/1471-2458/13/245/prepub
